# A Case of Pulmonary Actinomycosis With Concurrent Gastric Adenocarcinoma in an Older Adult

**DOI:** 10.7759/cureus.60180

**Published:** 2024-05-13

**Authors:** Ning Zhang, Changyi Liu, Lin Kang, Jianfeng Zhou, Wenjing Liu, Xuan Qu

**Affiliations:** 1 Department of Geriatrics, Peking Union Medical College Hospital, Peking Union Medical College, Chinese Academy of Medical Sciences, Beijing, CHN; 2 Department of Internal Medicine, Peking Union Medical College Hospital, Peking Union Medical College, Chinese Academy of Medical Sciences, Beijing, CHN; 3 Department of Oncology, Peking Union Medical College Hospital, Peking Union Medical College, Chinese Academy of Medical Sciences, Beijing, CHN; 4 Department of Geriatrics, Gansu Provincial Second People's Hospital, Lanzhou, CHN

**Keywords:** comprehensive geriatric assessment, treatment, older adult, gastric adenocarcinoma, pulmonary actinomycosis

## Abstract

Actinomycosis is a chronic granulomatous disease that can affect various parts of the body, including the head and neck, lungs, abdominal and pelvic cavities, and wounds. It is caused by different actinomycetes like *Actinomyces sherdii*, *Actinomyces glasii*, *Actinomyces cariosa*, *Actinomyces zurichensis*, and *Actinomyces europaea*. Reported infections caused by actinomycetes include pulmonary actinomycosis, pelvic and abdominal infections, bone or artificial joint infections, endocarditis, complicated urinary tract infections, and soft tissue abscesses. The combination of pulmonary actinomycosis with gastric cancer is exceptionally rare in clinical practice, and the presence of actinomycetal infection alongside tumors in elderly patients poses significant challenges in treatment. This article presents the diagnosis and treatment process of an elderly patient with pulmonary actinomycosis and gastric adenocarcinoma.

## Introduction

Actinomycetes, gram-positive anaerobic filamentous prokaryotic microorganisms belonging to the order Actinomycetales, display a radial colony shape. These opportunistic pathogenic bacteria are commonly present in various natural environments and can colonize different parts of the human or animal body, including the oral cavity, upper respiratory tract, gastrointestinal tract, and urogenital [1]. They are typically part of the normal oral flora, particularly in sites such as gingival crevices, tonsillar crypts, periodontal pockets, dental plaque, and cavities. Pathogenic bacteria, predominantly anaerobic actinomycetes like *Clostridium*, can cause infections in various body regions, with the head and neck being the most frequently affected (50-60%). Pulmonary actinomycosis is a rare suppurative infection caused by gram-positive, anaerobic filamentous bacteria from the Actinomycetaceae family. The clinical manifestations of this condition are varied, often mimicking other infectious lung diseases or malignancies. Due to the slow-growing and fastidious nature of the bacteria, diagnosing pulmonary actinomycosis can be difficult and may necessitate a biopsy for precise identification [2]. The co-occurrence of gastric cancer and pulmonary actinomycosis is exceedingly rare. This article presents a case study of pulmonary actinomycosis combined with gastric adenocarcinoma in an elderly patient, aiming to enhance clinicians’ comprehension of pulmonary actinomycosis and explore treatment strategies for gastric cancer in the presence of actinomycetal infection.

## Case presentation

**Figure 1:**
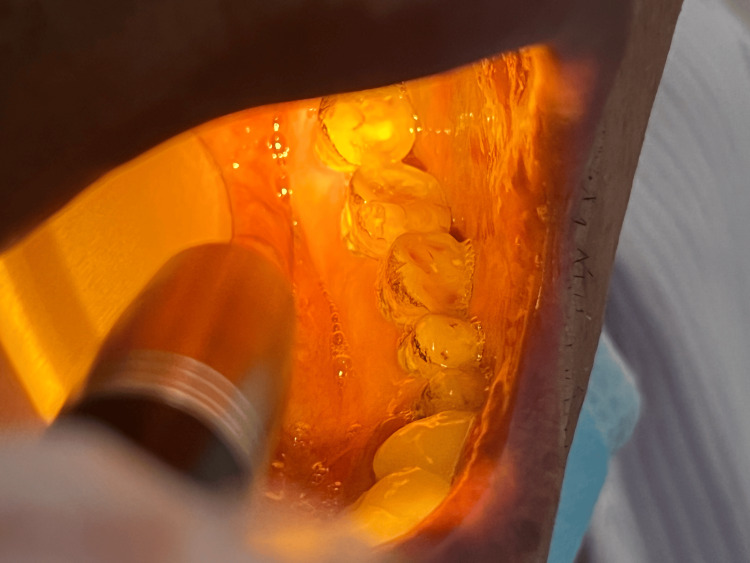
The physical examination revealed poor oral hygiene, characterized by red, swollen gums and extensive soft plaque on most teeth.

A 68-year-old male patient was admitted to the hospital due to persistent abdominal distension lasting over a month. The distension began after a heavy meal, without any accompanying abdominal pain, nausea, or vomiting. Additionally, a single occurrence of blood in the stool was noted. During the course of the disease, the patient occasionally coughed up a small amount of yellow, viscous sputum without fever, dyspnea, chest pain, or hemoptysis. The patient had lost 5 kg over the past three months. He was a farmer involved in year-round farming activities, including corn cultivation, pig rearing, and herbicide spraying with a tractor for three months a year. The patient had no previous history of chronic obstructive lung disease or bronchiectasis and had a history of alcohol consumption for nearly 40 years, averaging around 25 g/day, but had been abstinent for the last three years. He had also smoked for 40 years, ranging from two to 20 cigarettes per day, with a recent reduction to 10 cigarettes per day over the past three years, but had not quit smoking altogether. The patient’s abdominal distension progressively worsened, leading him to seek treatment at our hospital approximately 40 days after the initial onset of symptoms. Upon admission, the patient presented with a BMI of 29.0 kg/m^2^. He exhibited poor oral hygiene, characterized by red, swollen gums, extensive soft plaque on most teeth, and six dentures (Figure 1). A palpable lymph node measuring approximately 0.5 cm in diameter was identified on the left side of his neck. Auscultation revealed coarse breath sounds in both lungs without the presence of wet or dry rales. The heart rhythm was regular, and no murmurs were detected upon auscultation of each valve area. The patient’s abdomen was slightly distended and soft, lacking gastrointestinal shape or peristaltic waves, with normal bowel sounds at a rate of four to six times per minute. There were no signs of tenderness, rebound tenderness, or muscle tension. The liver area was tender, and Murphy’s sign was negative. A comprehensive geriatric assessment revealed a Katz ADL score of 6, a Lawton IADL score of 8, an NRS-2002 score of 3, dominant hand grip strength of 39.9 kg, and a walking speed of 1.5 m/s.

**Figure 2:**
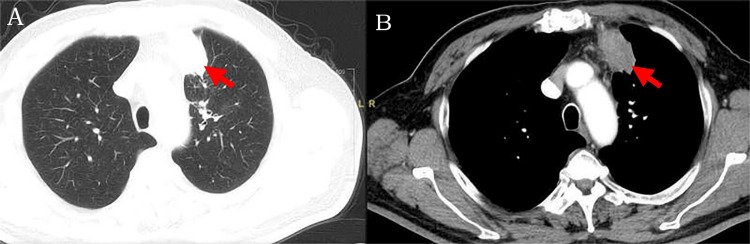
(A) CT lung window. (B) CT mediastinal window. The contrast-enhanced CT scan of the patient's chest revealed a 50.0 × 39.2 mm soft tissue density shadow in the left upper apical lobe, showing notable enhancement. This finding is highlighted in (A) and (B), indicated by red arrows.

**Figure 3:**
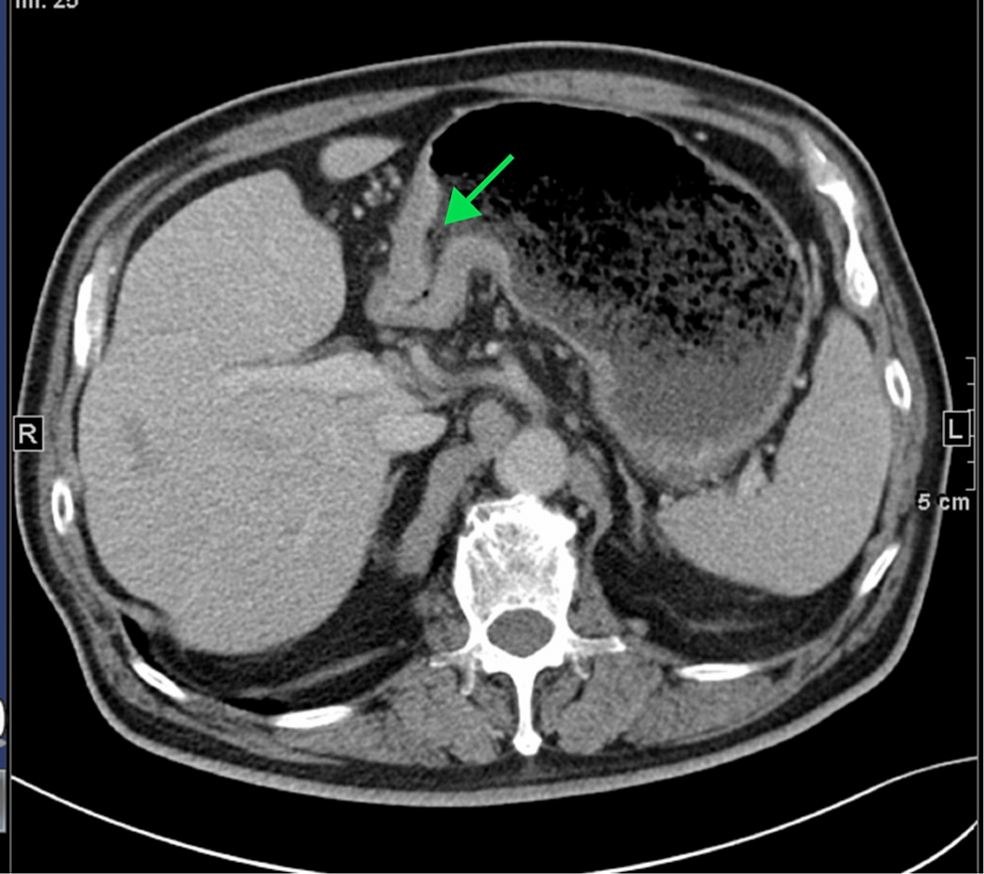
Enhanced CT of the patient’s abdomen revealed thickening and enhancement of the gastric antrum and gastric corner wall, as highlighted by the green arrow.

**Figure 4:**
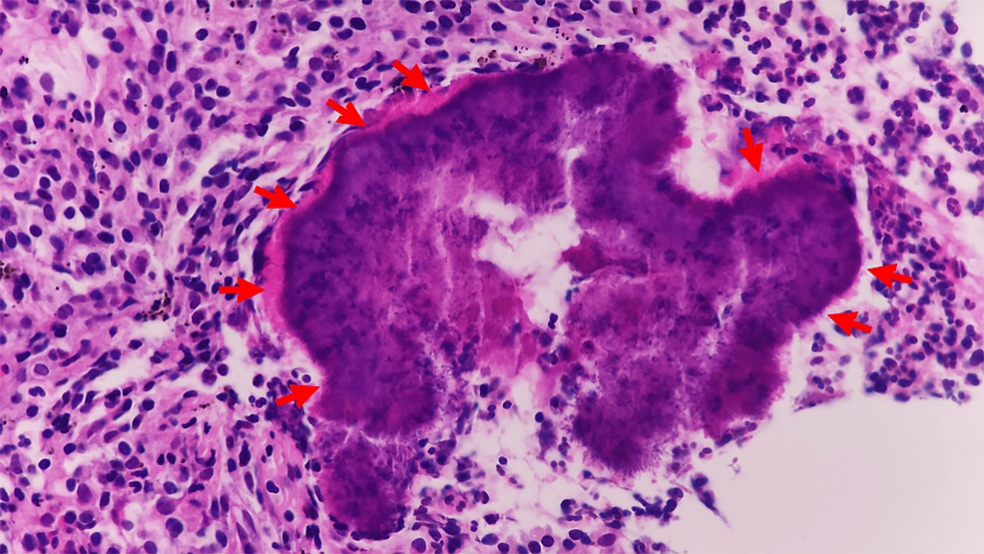
The pathology results from the CT-guided puncture biopsy of the mass in the left upper apical lobe revealed acute suppurative inflammation with infiltration of numerous lymphocytes and plasma cells. Actinomycete colonies, specifically sulfur granules (highlighted by red arrows), were identified, showing branched hyphae intertwined with a blue-purple center and radiating lines.

Following admission, a CT scan of the chest, abdomen, and pelvis identified irregular soft tissue density shadows in the posterior and anterior segments of the left upper apical lobe. These shadows, partially fused, displayed burrs and long cords at the edges. The larger shadow measured approximately 50.0 × 39.2 mm and exhibited significant enhancement (Figure 2). The lesion surrounded the posterior and anterior bronchial apex of the left upper lobe, extending to involve the left pleural roof and mediastinal pleura. Enlarged lymph nodes were also present in the left supraclavicular fossa, both hilum and mediastinum, indicating possible metastasis. Thickening with enhancement was noted in the gastric antrum and gastric corner walls (Figure 3), raising suspicion of malignancy. Plump lymph nodes were found in the liver-gastric space, with some showing enlargement suggestive of potential lymph node metastasis. Serum tumor markers showed elevated levels of CA19-9 at 277.0 U/ml (normal range: ≤34.0 U/ml) and CA242 at 88.9 U/ml (normal range: ≤25.0 U/ml). Following an improved gastroscopy, significant findings included gastric retention with a substantial amount of food residue in the mucus pool. Additionally, a circumferential ulcer-type mass was observed extending from the posterior wall of the lower curvature of the gastric body to the junction of the gastric angle and antral body. This mass was covered with debris and black blood clots. A biopsy of seven pieces was conducted, revealing tough (some brittle) tissue that easily bled. The lumen was narrow, but it allowed for endoscope passage. Pathology results indicated moderately to poorly differentiated adenocarcinoma. Immunohistochemistry results showed HP(-), PD-L1(22C3): CPS = 5, and Her-2(2+). A CT-guided puncture biopsy of the upper lobe of the left lung revealed chronic inflammation, widened alveolar septa with lymphoid tissue and plasma cell infiltration, and the presence of sulfur granules with irregular edges (Figure 4). Notably, hyphae extending out were observed, characteristic of an actinomycete mass.

**Figure 5:**
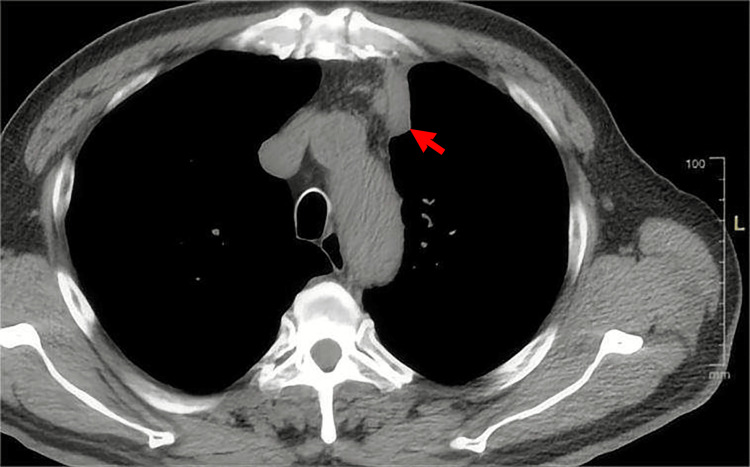
The patient’s follow-up chest CT scan revealed a notable decrease in soft tissue density in the left upper apical lobe compared to the initial admission scan, as indicated by the red arrow.

Following a lung tissue puncture biopsy under CT guidance and a mucosal biopsy under gastroscopy, the patient was diagnosed with pulmonary actinomycosis and gastric adenocarcinoma. Subsequent assessments revealed an ECOG score of 1 and a KPS score of 90. The patient initiated empiric treatment with amoxicillin and clavulanic acid. A follow-up chest CT two weeks later revealed multiple irregular soft tissue density shadows in the posterior and anterior segments of the left apical lobe, with the largest measuring approximately 39 × 19 mm. These shadows were significantly smaller than those observed on the initial CT scan upon admission (Figure 5). Furthermore, there were multiple enlarged lymph nodes in the left supraclavicular fossa, hilus, and mediastinum, with most being smaller in size compared to those observed on the admission CT scan. The patient commenced the first phase of the SOX regimen in conjunction with trastuzumab therapy for adenocarcinoma, while the anti-actinomycetal treatment was switched to oral amoxicillin. Nutritional support was enhanced by administering Nutrison fiber orally. The patient completed the initial course of the SOX regimen with trastuzumab successfully, experiencing no nausea, vomiting, shortness of breath, or hemoptysis, but did develop mild anorexia. Post-treatment, the white blood cell count and neutrophil levels remained within normal parameters.

## Discussion

The elderly male patient presented with an insidious onset of disease, with CT scans revealing a mass in the upper lobe of the left lung and abnormal thickening of the gastric antrum and corner wall. Subsequent procedures, including CT-guided puncture of the lung mass and a gastroscopic mucosal biopsy, led to the diagnosis of pulmonary actinomycosis along with gastric adenocarcinoma. Pulmonary actinomycosis, a chronic lung disease caused by actinomycetes, accounts for 15% of all cases of this condition [3]. Clinical symptoms are nonspecific and can be challenging to distinguish from other lung diseases, often resulting in misdiagnosis as lung cancer. The disease can occur at any age, with peaks in incidence between 11-20 years and 40-60 years being rare in children. Actinomycetes are commonly found in the normal flora of the human oral, gastrointestinal, and genitourinary tracts. Infections usually occur when there is tissue damage or disruption of the mucosal barrier, which can lead to invasion and the progression of disease [4]. Pulmonary actinomycosis is primarily caused by the aspiration of secretions or foreign bodies containing actinomycetes from the oropharynx or gastrointestinal tract, with subsequent spread to other areas. Risk factors for this condition include poor oral hygiene, increased aspiration risk, underlying lung diseases such as chronic pulmonary lung disease, bronchiectasis, and lung cancer, smoking, alcohol abuse, dental surgery, an immunocompromised state, and preexisting actinomycete infections [5]. The patient’s oral hygiene and history of heavy smoking are significant factors in the development of pulmonary actinomycosis.

Patients with pulmonary actinomycosis typically experience a slow progression of the disease, which can persist for several years, particularly in the elderly. Clinical symptoms and signs lack specificity, with common manifestations including cough, sputum, hemoptysis, chest pain, dyspnea, night sweats, fever, and weight loss. In cases where the pleura is involved, complications such as pleural effusion, empyema, superior vena cava obstruction syndrome, or pericardial effusion may arise. These symptoms closely resemble those of other respiratory conditions like pulmonary malignant tumors, pneumonia, and tuberculosis, often leading to misdiagnosis or missed diagnosis. Furthermore, involvement of the pleura can result in the formation of empyema and chest wall fistulas, with the discharge of pus containing sulfur particles. In instances where the mediastinum is affected, patients may experience difficulty breathing or swallowing, potentially leading to fatal outcomes [6]. Early detection of pulmonary actinomycosis is crucial to prevent complications resulting from a delayed or overlooked diagnosis. Clinical studies indicate that the accuracy of diagnosing this condition is typically below 4-7%, with approximately 25% of cases initially misdiagnosed as malignant tumors [7]. Diagnosis of pulmonary actinomycosis poses challenges, with differential diagnosis relying on factors such as positive culture results, the presence of sulfur-containing pus in infected tissue, correlation with clinical and imaging findings, and response to antibiotic therapy. Specific laboratory tests for diagnosing pulmonary actinomycosis are lacking, and nonspecific indicators include anemia, leukocytosis, elevated levels of CRP, and erythrocyte sedimentation rate. While imaging studies are not definitive for diagnosis, they play a key role in assessing lesion location and severity for biopsy and monitoring treatment response. Imaging findings are typically nonspecific, with chest X-rays often revealing masses or consolidations with cavities distributed across the lobes. CT scans commonly show central necrotic masses at the lung peripheries, sometimes with lobular or spicular patterns, along with cavitation, atelectasis, and mediastinal or hilar lymph node enlargement. Additionally, pleural thickening and effusion may be present [8]. Pulmonary actinomycosis can sometimes appear as soft tissue density in the lung with high metabolic activity on PET-CT scans, which can initially be mistaken for lung cancer [9]. When there is suspicion of pulmonary actinomycosis, PET-CT may not be useful in distinguishing between lung cancer and benign lesions [10].

Pulmonary actinomycosis has been documented to coexist with lung cancer in the literature [11]. The patient’s thoracoabdominal and pelvic contrast-enhanced CT scan revealed multiple enlarged lymph nodes in the hilus and mediastinum, alongside a long-standing history of heavy smoking, indicating risk factors for lung cancer. Vigilance is advised for the potential simultaneous occurrence of lung cancer. Following a two-week course of intravenous anti-infective treatment with amoxicillin and clavulanic acid, a subsequent chest CT scan showed a significant reduction in the mass in the upper lobe of the left lung, as well as shrinkage in the left supraclavicular fossa, hilar, and mediastinal lymph nodes, further supporting the diagnosis of actinomycosis in the lung. Close monitoring of chest imaging is essential to track changes in lung lesions.

Actinomycosis, also known as “the great masquerade,” can present as systemic manifestations that mimic various types of malignancies in different parts of the body. For example, vertebral and pulmonary actinomycosis can resemble metastatic lung cancer, while ovarian, rectal, pelvic, and pancreatic actinomycosis may be mistaken for malignancies in their respective organs [12-17]. In the case of primary gastric actinomycosis, patients may experience symptoms suggestive of gastric outlet obstruction, leading to misdiagnosis as gastric cancer or lymphoma initially. Although rare, primary gastric actinomycosis should be considered in cases of diffuse gastric wall thickening or submucosal tumor-like lesions seen on radiologic imaging or gastroscopy [18]. Additionally, gastric actinomycosis can be asymptomatic and incidentally detected during gastroscopy [19].

A definitive diagnosis of pulmonary actinomycosis typically necessitates isolating actinomycetes directly from specimens. Given the challenging culture conditions and high technical demands for anaerobic bacteria, direct culturing of pathogenic bacteria is often difficult, with positive results achieved only in rare instances. The primary method for confirming the diagnosis of pulmonary actinomycosis is through pathological examination of tissues obtained via bronchoscopy biopsy, percutaneous lung puncture, and surgical resection, among other procedures. Moreover, advancements in molecular biology detection technology, such as microbial second-generation sequencing, have emerged as valuable tools for diagnosing infectious diseases [20]. Treatment of actinomycosis usually involves initiating penicillin at a dosage of 18-24 million U/day intravenously for a period of two to six weeks, followed by oral amoxicillin (three to six months for mild cases and six to 12 months for severe cases). Tetracycline, erythromycin, and clindamycin can serve as alternative options if needed. In cases with a poor response to anti-infective agents, the presence of an abscess or empyema, a sinus or fistula, life-threatening hemoptysis, etc., surgical intervention may be necessary, with continued anti-infective therapy post-surgery [7].

This patient presented with gastric adenocarcinoma and significant gastric retention. Following the initial management of the pulmonary actinomycete infection, specific treatment for the tumor should be initiated. The gastric cancer pathology of the patient reveals moderately poorly differentiated adenocarcinoma with molecular characteristics showing Her-2++; Fish+, PD-L1(22C3): CPS = 5, EBER ISH(-). The HER2-positive rate among Chinese gastric cancer patients ranges from 8.8% to 12% [21]. In cases of Fish+ gastric adenocarcinoma, the addition of the anti-HER2 monoclonal antibody trastuzumab has been shown to enhance treatment efficacy [22]. A comprehensive geriatric assessment indicated good physical function in the patient. After careful consideration of the risks and benefits, a treatment plan consisting of reduced-dose SOX double-drug chemotherapy in combination with trastuzumab was determined. Close monitoring of the absolute neutrophil count is essential during the course of treatment. Literature reports suggest that patients with lung cancer may be susceptible to secondary actinomycete infections during treatment [23,24], underscoring the importance of continued anti-actinomycete therapy and vigilant monitoring of the patient’s chest imaging changes.

The existing literature does not report any previous cases of concurrent pulmonary actinomycosis and gastric cancer. This case study represents the first documented instance of gastric cancer complicated by pulmonary actinomycosis. There is currently no established protocol for treating both conditions in elderly patients. Maintaining the functionality of older individuals is a key goal in geriatric medicine. In this case, a personalized treatment plan should be developed through collaborative decision-making involving the medical team and the patient, considering factors such as overall health, functional status, willingness to undergo treatment, and life expectancy. After initial anti-actinomycete therapy led to a significant reduction in lung lesions within two weeks, the patient opted for active treatment for gastric cancer. A cautious approach was taken to address the actinomycete infection and initiate gastric cancer treatment simultaneously. Elderly patients often present with multiple concurrent diseases, posing challenges in treatment planning. Developing optimal treatment plans in such cases requires careful consideration of various factors by a geriatric interdisciplinary team, including patient preferences and the risks versus benefits of treatment. This complex task presents challenges and opportunities for geriatricians, highlighting the importance of clinical practice and research in finding solutions.

## Conclusions

Pulmonary actinomycosis, a chronic suppurative lung disease caused by actinomycete infection, has a low incidence rate and poses challenges in obtaining microbiological evidence. In the early stages, it can often be misdiagnosed as lung cancer, pulmonary mycosis, tuberculosis, or lung abscess. Particularly in elderly patients with a history of smoking and poor oral hygiene, distinguishing lung masses seen on imaging from pulmonary actinomycosis is crucial. When managing patients with concurrent pulmonary actinomycosis and gastric cancer, it may be advantageous to not only provide effective anti-infective therapy but also customize treatment strategies for the tumor based on its histological subtype, molecular profile, and the patient’s overall health status. Continuous close follow-up observation is necessary to assess the long-term prognosis and clinical outcome of this patient.
